# Mitochondrial Glutathione: Recent Insights and Role in Disease

**DOI:** 10.3390/antiox9100909

**Published:** 2020-09-24

**Authors:** Montserrat Marí, Estefanía de Gregorio, Cristina de Dios, Vicente Roca-Agujetas, Blanca Cucarull, Anna Tutusaus, Albert Morales, Anna Colell

**Affiliations:** 1 Department of Cell Death and Proliferation, Institute of Biomedical Research of Barcelona-Spanish Council of Scientific Research, August Pi i Sunyer Biomedical Research Institute, 08036 Barcelona, Spain; estefania.degregorio@iibb.csic.es (E.d.G.); cristina.dedios@iibb.csic.es (C.d.D.); vicente.roca@iibb.csic.es (V.R.-A.); Blanca.cucarull@iibb.csic.es (B.C.); anna.tutusaus@iibb.csic.es (A.T.); 2 Departament de Biomedicina, Facultat de Medicina, Universitat de Barcelona, 08036 Barcelona, Spain; 3 Barcelona Clinic Liver Cancer Group, Liver Unit, Hospital Clínic, Network Center for Biomedical Research in Hepatic and Digestive Diseases (CIBEREHD), 08036 Barcelona, Spain; 4 Network Center for Biomedical Research in Neurodegenerative Diseases (CIBERNED), 08036 Barcelona, Spain

**Keywords:** mitochondria, glutathione, oxidative stress, antioxidant, steatohepatitis, Alzheimer, diabetic nephropathy, aging

## Abstract

Mitochondria are the main source of reactive oxygen species (ROS), most of them deriving from the mitochondrial respiratory chain. Among the numerous enzymatic and non-enzymatic antioxidant systems present in mitochondria, mitochondrial glutathione (mGSH) emerges as the main line of defense for maintaining the appropriate mitochondrial redox environment. mGSH’s ability to act directly or as a co-factor in reactions catalyzed by other mitochondrial enzymes makes its presence essential to avoid or to repair oxidative modifications that can lead to mitochondrial dysfunction and subsequently to cell death. Since mitochondrial redox disorders play a central part in many diseases, harboring optimal levels of mGSH is vitally important. In this review, we will highlight the participation of mGSH as a contributor to disease progression in pathologies as diverse as Alzheimer’s disease, alcoholic and non-alcoholic steatohepatitis, or diabetic nephropathy. Furthermore, the involvement of mitochondrial ROS in the signaling of new prescribed drugs and in other pathologies (or in other unmet medical needs, such as gender differences or coronavirus disease of 2019 (COVID-19) treatment) is still being revealed; guaranteeing that research on mGSH will be an interesting topic for years to come.

## 1. Introduction

**Figure 1:**
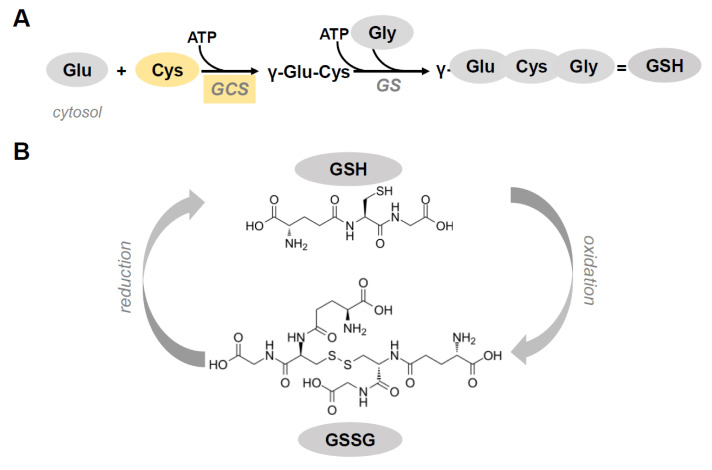
(**A**) Glutathione (GSH) synthesis in cytosol. GSH is synthesized by the concerted action of two enzymes. The first reaction is the formation of γ-glutamylcysteine from glutamate and cysteine by the enzyme γ-glutamylcysteine synthetase (GCS). The last step in GSH synthesis is regulated by GSH synthetase (GS). GCS and Cysteine (in yellow) are the limiting factors in GSH synthesis. (**B**) Chemical structure of reduced (GSH) and oxidized (GSSG) glutathione.

Glutathione (γ-l-glutamyl-l-cysteinyl-glycine, GSH), the most abundant thiol found in virtually all cells, is a tripeptide synthesized in the cytosol by two Adenosine triphosphate (ATP)-consuming enzymatic reactions. The first reaction, the formation of γ-glutamylcysteine from glutamate and cysteine by the enzyme γ-glutamylcysteine synthetase (GCS), is rate-limiting due to the usually low availability of cysteine. Of note, the inhibition of this reaction by GSH constitutes a regulatory step for maintaining a proper GSH concentration intracellularly [1,2]. The last step in GSH synthesis, regulated by GSH synthetase (GS), requires γ-glutamylcysteine and glycine as substrates (Figure 1A) [3]. The high concentration of GSH, reaching millimolar levels (1–10 mM) within cells and micromolar levels (10–30 μM) in plasma, and its low redox potential (E^′^_0_ = −240 mV) make GSH a perfect cellular redox buffer [4,5,6]. 

GSH has two characteristic structural properties: a γ-glutamyl bond and a sulfhydryl group, which give GSH its distinctive qualities—stability and reductive power. GSH participates in transhydrogenation reactions necessary for the formation and maintenance of the reduced state of sulfhydryl groups of other molecules, proteins, and enzymes. GSH acts as a reducing agent in various reactions, for example, the formation of deoxyribonucleotides and in the reduction of dehydroascorbate to ascorbate. GSH also participates in the detoxification of xenobiotics, which, after interacting with glutathione, are excreted in the form of mercapturic acid into urine or feces [7]. Moreover, in the metabolism of prostaglandins, leukotrienes, steroids, and melanin byproducts of GSH are formed after conjugation with the endogenous metabolites. It has been reported that the γ-glutamyl part of GSH participates in the transport of amines, peptides, and aminoacids (especially cystine and other neutral amino acids) [8,9].

The thiol group of the aminoacid cysteine in the backbone of GSH is responsible for its antioxidant capacity. This redox-active thiol residue becomes oxidized when GSH reduces target molecule to form glutathione disulfide (GSSG) (Figure 1B). The GSSG/GSH redox couple, being the most abundant in the cells, can interact with other antioxidant redox couples to properly balance the redox environment in the cells [10]. 

The fact that GSH can directly eliminate free radicals and reduce H_2_O_2_ is a first line of defense against reactive oxygen species (ROS). On the other hand, a second line of defense is formed by glutathione-dependent enzymes that detoxify by-products generated by ROS and therefore help prevent ROS propagation. Thus, GSH participates as a co-factor in several reactions, including the elimination of peroxides by GSH peroxidases (GPx), the covalent addition to protein cysteines (Protein-SSG) predominantly performed by glutaredoxin, and the detoxification of electrophiles by S-glutathionylation (GS-R) formation as catalyzed by the enzymes glutaredoxin and glutathione S-transferase (GST) [4,11,12]. In addition, an increase in protein S-glutathionylation through the formation of sulfenic acid or nitrosated cysteine intermediates, or by changes in the GSH redox, is associated to conditions such as hypertension, ischemia-reperfusion, and tachycardia where oxidative and nitrosative stresses are present. The presence of sulfenic acid or nitrosated cysteine intermediates promotes reversible S-glutathionylation of strategic proteins involved in cell signaling, ion transport, energy production, and cell death. In fact, recent studies indicate that the S-glutathionylation-deglutathionylation cycle cooperates with other post-translational mechanisms in regulating signal transduction, inflammation, metabolism, and apoptosis; therefore, it is emerging as an important post-translational modification [13].

GSSG can rapidly be recycled back to GSH by nicotinamide adenine dinucleotide phosphate (NADPH)-dependent glutathione reductase (GR) in key organelles and the cytosol such that the glutathione pool is largely reduced with little GSSG being present [10]. Thus, measuring the ratio GSSG to GSH is an indicator of cellular oxidative stress. GSH and GSSG are found outside cells, but normally at very low concentration—100 to 1000 times less than intracellular GSH. Extracellular GSH is thought to function in detoxification along with providing protection against oxidants.

GSH is synthesized exclusively in the cytosol; nonetheless it is found present at different intracellular organelles such as the endoplasmic reticulum (ER), nucleus, and mitochondria. This compartmentation results in separate redox pools of GSH, where it performs specific functions [2]. The independence of these separate GSH pools, for example, is supported by the observation that treatment with L-buthionine-SR-sulfoximine (BSO), an inhibitor of GSH synthesis, does not result in a complete reduction in the nuclear GSH, as compared to cytosolic GSH [14]. Nuclear GSH is responsible for the maintenance in the reduced state of protein sulfhydryls crucial for DNA expression and repair. In addition, in the active phases of cell proliferation, the nucleus accumulates GSH to much greater concentrations than those present in the cytoplasm [15]. 

GSH mostly exists in its reduced form in cytosol, nucleus, and mitochondria, while in the endoplasmic reticulum (ER) the ratio GSH/GSSG is in the range from 1:1 to 3:1 [16], to properly favor the correct folding of proteins that have essential disulfide bonds. Thus, in the lumen of the ER, there is a substantially higher concentration of GSSG, as compared with the rest of the cell which allows the formation of native protein disulfide bonds and also the isomerization of non-native disulfide bonds. Both reactions, which can rarely be formed in the cytosol because of the high concentration of GSH, are mainly catalyzed by protein disulfide isomerase (PDI) [16,17].

## 2. Mitochondrial Glutathione

**Figure 2:**
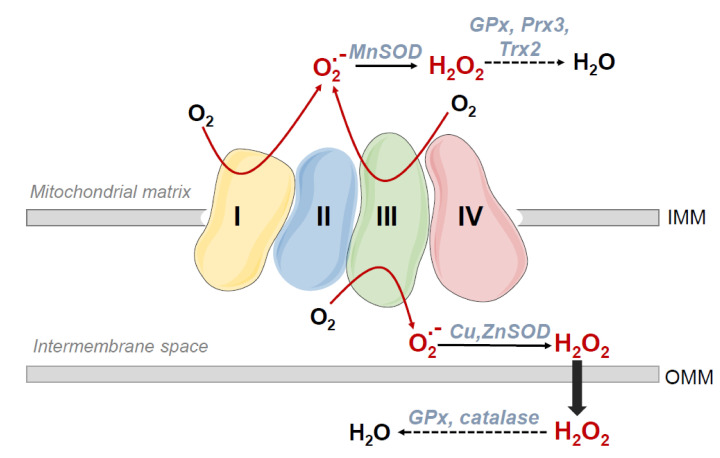
Sources of reactive oxygen species (ROS) in the electron transport chain. During mitochondrial respiration partial reduction reactions occur, mainly from complexes I and III that cause the accumulation of superoxide and hydrogen peroxide mainly in the mitochondrial matrix.

Within the cells, mitochondria are not only the primary site of oxygen consumption, but also the major source of reactive oxygen species (ROS), most of them originating at the electron transport chain (ETC). For a recent review on the subject, see [18]. During mitochondrial respiration some “leakiness”, or partial reduction reactions occur, mainly from complexes I and III even under physiologic conditions (Figure 2). This leakiness causes the release of superoxide and hydrogen peroxide mostly to the mitochondrial matrix [19,20]. In fact, it has been estimated that superoxide concentration is five- to ten-fold higher in the matrix than that in the cytosol [21]. 

Therefore, mitochondria need constant protection from the toxic action of ROS as they constitute an important source. Low molecular weight antioxidants, such as GSH, vitamin E, or ubiquinone, as well as enzyme defense systems, are responsible for providing this protection. Concerning GSH, it was proposed that depletion of the mitochondrial GSH pool frequently correlated better to toxic cell death than overall loss of intracellular GSH. In addition, the mitochondrial GSH was more resilient to exhaustion, upon inhibition of GSH synthesis, than other intracellular GSH pools [22,23].

Mitochondrial GSH (mGSH) regulates mitochondrial ATP production by modifying critical protein sulfhydryl redox states that consequently influence nicotinamide adenine dinucleotide (NADH) and flavin adenine dinucleotide (FADH2) generation and electron flow in the electron transport chain (ETC). Principally, Complex I contains central redox active thiols that can be reversibly glutathionylated to regulate electron flux in the event of enhanced oxidative stress [3,24,25]. mGSH acts in concert with other antioxidant enzymes such as GPx1 and GPx4, GSTs, glutaredoxin-2, and ATP binding cassette transporters to maintain mitochondrial function [26,27]. 

**Figure 3:**
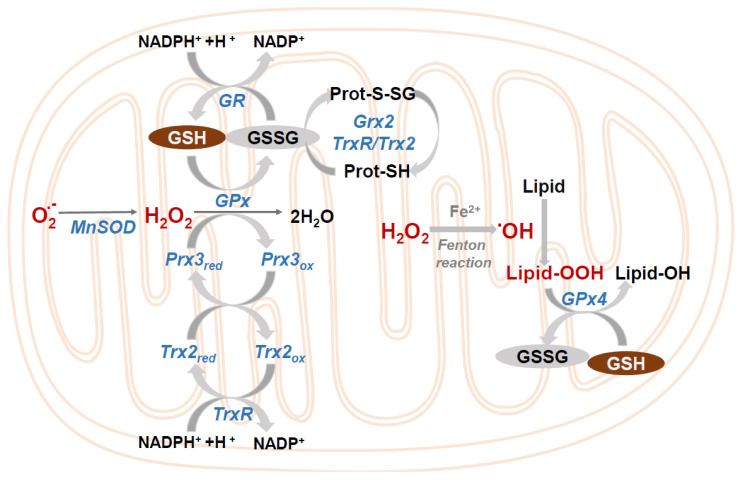
Mitochondrial control of ROS. Different reactions that take place in the mitochondria to eliminate superoxide and hydrogen peroxide. GSH peroxidase (GPx); GSSG-reductase (GR); GSSG, glutaredoxin-2 (Grx2); Mn-dependent superoxide dismutase (MnSOD); thioredoxin-2 (Trx2); Trx-reductase (TrxR); peroxiredoxin-4 (Prx3).

The mitochondrial GSH pool is maintained in the reduced state by GR that couples GSSG reduction to the matrix NADP+/NADPH pool (Figure 3) [3,23]. The physiological relevance of keeping mitochondrial oxidative stress and redox status is paramount, as evidenced by the fact that the knock-out mice of the mitochondrial enzymes GPx4 (also known as phospholipid hydroperoxide glutathione peroxidase) or TrxR2 are embryonic lethal [28]. GPx4, using mGSH as a cofactor, is a lipid repair enzyme critical for the reduction of the lipid hydroperoxides formed by the Fenton reaction. This reaction occurs when excess iron, in the ferrous form (Fe^2+^), interacts with H_2_O_2_ forming as a consequence the hydroxyl radical, a short-life but highly reactive specie that promotes oxidative DNA damage, denaturation of proteins, and lipid peroxidation [29,30] (Figure 3). Failure to control the excess iron and ROS can lead to ferroptosis, a programmed form of cell death characterized by massive lipid peroxidation. In fact, the sole inhibition of GPx4 can trigger ferroptosis [31].

mGSH together with the thioredoxin system, in particular mitochondrial thioredoxin 2/thioredoxin reductase 2 (Trx2/TrxR2), maintain thiol redox status within mitochondria [32]. In addition, peroxiredoxins (Prx), a family of thiol-specific peroxidases that reduce lipid hydroperoxides and H_2_O_2_ [33] rely on thioredoxins (Trxs) as their hydrogen donor. Prx3, exclusively located in mitochondria, depends on the Trx2/TrxR2 system for its reduction (Figure 3). Of importance, depletion of mGSH causes interference with either the GSH system or the Trx2 system due to oxidation of the dithiol on the active site of Trx2, thus sensitizing to ROS-induced cell death. These data enhance the non-redundant functions in the protection against oxidative stress of the GSH and Trx2 systems [34]. 

Notably, while protein S-glutathionylation is very common throughout the cell, within the mitochondria the proteins are greatly predisposed to reversible S-glutathionylation. In fact, mitochondria contain a large number of proteins, from those involved in energy metabolism, solute transport, ROS production, to inducers of apoptosis, antioxidant defense, and those responsible for mitochondrial dynamics, that are targeted by S-glutathionylation. In addition, defects in the reactions responsible for the conjugation and elimination of GSH in mitochondrial proteins may have direct pathological consequences [12,35].

### Transport of GSH across the Inner Mitochondrial Membrane

**Figure 4:**
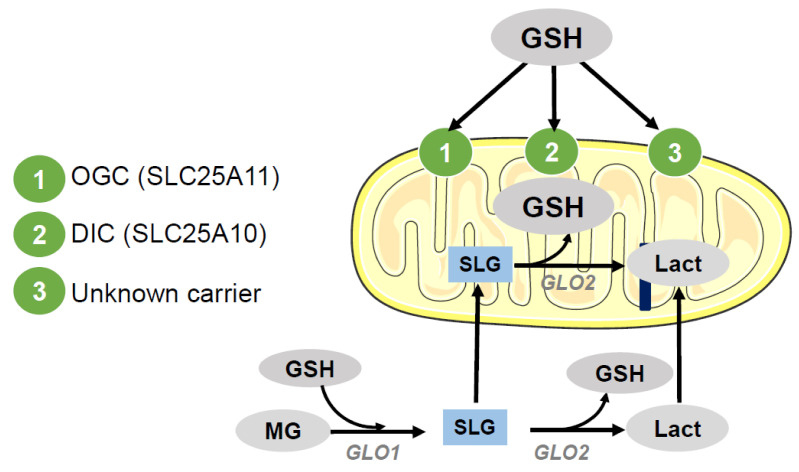
GSH transport to mitochondria. Once GSH is synthesized in the cytosol it can be transported to the mitochondria by specific IMM carriers: 2-oxoglutarate carrier (OGC; SLC25A11) and dicarboxylate carrier (DIC; SLC25A10), although the presence of unknown carriers cannot be discarded at present. In addition, S-d-lactoylglutathione (SLG), a stable intermediate product of the glyoxalase (GLO1) system which catalyzes the conversion of methylglyoxal (MG) into d-lactic acid (Lact), can enter the mitochondria and by the action of mitochondrial glyoxalase II (GLO2), be hydrolyzed to lactate and mGSH.

Several aspects indicate that a carrier-mediated process accounts for the transport of GSH into mitochondria. Among them, the absence of GSH synthesizing enzymes in mitochondria, the negative charge of GSH at physiological pH and the negative potential of the inner mitochondrial membrane are relevant, despite similar concentrations of GSH found in cytosolic and mitochondrial compartments. In fact, to date, the two reported transporters of mGSH capable of catalyzing the uptake of GSH into the mitochondrial matrix are anion carriers, members of the mitochondrial carrier family (SLC25), the mitochondrial dicarboxylate carrier (DIC; SLC25A10), and the 2-oxoglutarate carrier (OGC; SLC25A11). These mGSH carriers, which mediate an electroneutral exchange, have been mostly described in liver and kidney cells [36,37,38,39,40]. Of pathological interest, the OGC transport of mGSH in liver is dependent on membrane dynamics, since cholesterol accumulation in the inner mitochondrial membrane (IMM) results in decreased mGSH transport, and mitochondrial membrane fluidification restores mGSH uptake [41]. Therefore, in addition to regulating mGSH transport, cholesterol modulates susceptibility to oxidative stress and cell death. In this way, cholesterol is set as an important target in the pathophysiology of various diseases as diverse as steatohepatitis (SH) or Alzheimer’s disease. [42,43,44,45]. Additional studies have suggested that there are intraorgan differences in the transport of mGSH [38,41] and that DIC and OGC are only partially responsible for GSH uptake in rat liver mitochondria [46]. This implies that other putative mGSH carriers are still unknown (Figure 4).

In contrast, a recent study by Booty et al. [47] using the *Lactococcus lactis* system for overexpression and characterization of members of the mitochondrial carrier family [48] showed no detectable transport of mGSH by DIC and OGC carriers. Thus, confirmatory studies in either scenario are needed to better define mGSH carriers in different cell types and organs.

Interestingly, an additional source of mGSH is the one obtained through S-d-lactoylglutathione (SLG), a stable intermediate product of the glyoxalase system which catalyzes the conversion of methylglyoxal into d-lactic acid [49]. SLG can enter the mitochondria, and by the action of mitochondrial glyoxalase II (GLO2), be hydrolyzed to lactate and mGSH without the need for ATP [49] (Figure 4). However, it has not yet been determined the amount of mGSH obtained by SLG and the importance of this pathway as compared to mGSH carriers. Related to this, it has also been described in vitro that GLO2, using SLG as a substrate, can induce the S-glutathionylation of metabolic enzymes of different cellular compartmentalization, in particular malate dehydrogenase, cytochrome b, and complex I from the mitochondria [12,35,50,51], although the relevance of this observations rests to be determined in vivo. Moreover, it has been recently proposed that S-glutathionylation of proteins in response to the oxidation of GSH is a means for the inhibition of catabolic pathways leading to a reduction in ROS generation, and consequently as a mechanism for desensitization of H_2_O_2_ signals [35]. Hence, protein S-glutathionylation could act as a post-translational modification to associate energy metabolism to redox signaling [12,35,51].

## 3. mGSH and Cell Death

Cell death is a regulated process and has been evolutionarily conserved in different species from embryogenesis to the maintenance of homeostasis in adult tissues. The different types of cell death are defined by morphological criteria and occur following different pathways. The two most studied and characteristic modes of mammalian cell death are apoptosis and necrosis. For a more extensive review on the molecular mechanisms of cell death, see [52]. 

**Figure 5:**
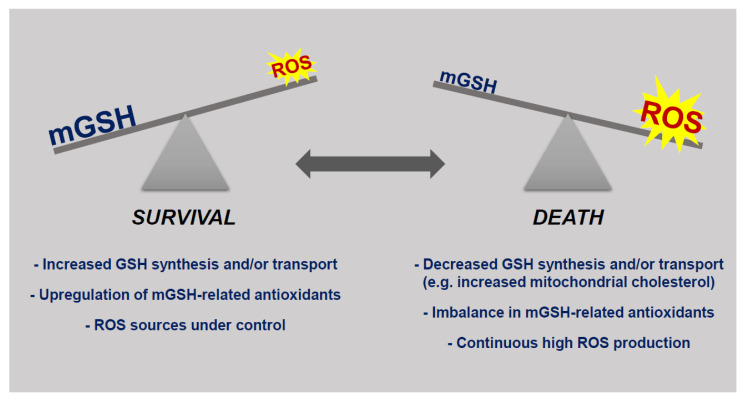
The balance between mGSH levels and reactive oxygen species (ROS) present in the mitochondrial milieu determines cellular susceptibility to death stimuli. Under physiological conditions or even in the continuous presence of increased ROS production survival is guaranteed due to upregulation of GSH-related antioxidant mechanisms. Mitochondrial death only arises when the production of ROS is high and/or the levels of mGSH are compromised due to minor synthetic capabilities or problems with mGSH transport.

An integral part of apoptotic and necrotic cell death is mitochondrial ROS production. Consequently, antioxidants such as mGSH combat oxidative stress and increase cell viability in multiple experimental models [53]. A rigorous balance between mitochondrial ROS generation and inactivation, under physiological conditions, is necessary for the maintenance of cellular functions and viability. Loss of this balance can lead to cell death [54] (Figure 5).

The availability of GSH is limiting for the activity of GSH-dependent antioxidant defense systems. In the context of mGSH, there is ample evidence of its importance for cell survival. In general, mitochondrial thiols have been shown to act as regulators of cell death pathways [55,56], and, in particular, it has been reported that mGSH depletion is a trigger for cell death. Consequently, promotion of cell death correlates more closely to the extent of depletion of mGSH rather than the changes in the GSH cytoplasmic pool in diseases or treatments that deplete cellular GSH [25,40]. Selective mGSH depletion is able to sensitize to cell death by promoting oxidative stress and nitrosative stress [57]. Furthermore, mitochondrial GSSG resulting from GSH oxidation must be efficiently reduced back to GSH by mitochondrial GR. This, in addition, requires the availability of mitochondrial NADPH, which also provides reducing equivalents for TrxR2 and is consequently vital for the functioning of the thioredoxin and peroxyredoxin systems. Mitochondrial NADP^+^-dependent isocitrate dehydrogenase (IDPm) and the proton-translocating nicotinamide nucleotide transhydrogenase located in the IMM are the enzymes responsible for NADPH regeneration [58]. Therefore, as expected, modulation of the activity of both enzymes is inversely related to cellular apoptosis susceptibility [59,60].

Of interest, in addition to ROS formation, iron overload followed by stimulation of mitochondrial lipid peroxidation may also induce a general suppression of mitochondrial metabolism. Important mitochondrial functions, such as respiration and oxidative phosphorylation, mitochondrial membrane potential (Δψ), and mitochondrial Ca^2+^ buffering capacity can be altered by lipid peroxides [61,62,63,64]. In addition, mitochondrial lipid peroxidation derivatives can damage membranes by altering their barrier function by either directly interacting with membrane proteins and/or indirectly with lipid moieties [65]. 

In recent years, mitochondria have been recognized as regulators of cell death by apoptosis and via necrosis. In aerobic cells, in addition to ATP production, mitochondria play an essential role in the regulation of intracellular Ca^2+^ homeostasis. Importantly, a potentially harmful effect of ROS production in mitochondria is facilitation of Ca^2+^-dependent mitochondrial permeability transition (MPT), a step that contributes to cell death [54]. Thus, oxidative stress significantly sensitizes mitochondria to MPT induction. In fact, it has been reported that mitochondrial-generated ROS are directly involved in the induction of MPT [66]. Consequently, both oxidative stress and impaired Ca^2+^ homeostasis promote mitochondrial-mediated cell damage. 

MPT leads to mitochondrial failure. If there is substantial ATP depletion necrosis will occur, and apoptosis will take place if there is activation of caspases and MPT only ensues in a subpopulation of mitochondria, but the remaining organelles are still able to produce ATP and preserve mitochondrial membrane potential. Moreover, studies have shown that membrane-bound GST1 in the inner mitochondrial membrane could interact with MPT regulator proteins, such as adenine nucleotide translocator (ANT) and/or cyclophilin D, and could contribute to oxidant-induced MPT pores [67]. 

## 4. mGSH in Pathological Settings

### 4.1. Alcoholic Liver Disease

Excessive alcohol exposure leads to alcoholic liver disease (ALD), one of the most serious consequences of chronic alcohol abuse, and a predominant cause of liver-related morbidity and mortality worldwide. The increased production of ROS observed after acute or chronic ethanol treatment reduces cellular antioxidant levels and enhances oxidative stress in many tissues, especially in hepatic tissue. Ethanol-induced oxidative stress plays an important role in the mechanisms by which ethanol causes liver damage [68]. The loss of oxidative phosphorylation and the defective ATP generation observed in mitochondria after ethanol treatment indicate that mitochondria are specific targets of the toxic effects of ethanol. Studies in animal models of chronic ethanol feeding have shown mitochondrial functional modifications, whereas patients with alcoholic steatohepatitis (ASH) had mitochondria with morphological and functional abnormalities [69,70,71]. mGSH becomes depleted by alcohol intake [72,73]. Of interest, alcohol feeding has been shown to sensitize hepatocytes to tumor necrosis factor (TNF), an important mediator of ASH. This sensitization to TNF is due to the limitation of mGSH, as a result of the ethanol-induced mitochondrial cholesterol increase that alters membrane-order parameter and partially inactivates the mGSH carrier [41,74]. In vitro, pharmacologic lessening of mGSH sensitizes hepatocytes to tumor necrosis factor (TNF)-mediated cell death, which parallels the findings observed after alcohol intoxication [75]. Selective decrease in mGSH, but not in cytosolic GSH, after alcohol intake has also been reported by other groups [76,77,78]. Alcohol feeding causes the accumulation of cholesterol in mitochondrial membranes, and subsequent mGSH depletion, by stimulating the expression of the mitochondrial cholesterol carrier steroidogenic acute regulatory protein (StARD1) [79]. GSH precursors are unproductive in refilling mGSH levels due to the primary defect in cytosolic GSH transport into mitochondria, despite a significant increase in cytosolic GSH. In contrast, S-adenosyl-l-methionine (SAM) administered to rats fed alcohol chronically has been shown to be able to replenish mGSH levels due to its effect on the normalization of the physical properties of the IMM [72]. Of note, subsequent studies have revealed that the depletion of mitochondrial SAM precedes that of mGSH and occurs independently of alcohol-mediated disturbances in membrane dynamics. Therefore, refuting that alcohol causes an inherent defect in mSAM transport, and suggesting that after alcohol feeding early reduction of mSAM contributes to changes in mitochondrial membrane dynamics and the consequent decrease in mGSH [80]. Interestingly, ethanol metabolism through CYP2E1 (cytochrome P450 2E1) is a fundamental step that contributes to hepatic oxidative stress. CYP2E1 is induced after chronic ethanol ingestion and because is a poorly coupled enzyme formation of ROS arises even without substrate. In fact, liver human liver hepatocellular carcinoma cell line (HepG2) cells overexpressing CYP2E1, where an increase in cellular ROS is detected, display a significant rise in cellular GSH levels (30%) that is due to an increased rate of GSH synthesis and an enhanced expression of GCS heavy subunit (GCS-HS) mRNA, the rate-limiting enzyme in GSH synthesis [81]. Moreover, these cells also display an enhanced expression of alpha and microsomal GST and of catalase [82]. These cellular adjustments afford protection against prooxidants and reflect an adaptive response by the cells in front of CYP2E1-derived oxidative stress. Thus, it is conceivable that despite the initial adaptation of hepatic cells to compensate with enhanced GSH synthesis and antioxidant capacity the surge of ROS inherent to ethanol metabolism [81,83], mGSH levels cannot be fully restored because of ethanol-induced rise of mitochondrial cholesterol and the consequent reduced mGSH transport to the mitochondria [41].

### 4.2. Non Alcoholic Fatty Liver Disease

Non-alcoholic fatty liver disease (NAFLD) exists as a continuum of disease ranging from extreme buildup of fat within the hepatic parenchyma (simple steatosis), inflammation (non-alcoholic steatohepatitis, NASH) to fibrosis, cirrhosis, end-stage liver disease, and there is also an increased risk of hepatocellular carcinoma (HCC). The main risk factors for NAFLD are obesity, along with type 2 diabetes, and concurrently, NAFLD is also a risk factor for the occurrence of type 2 diabetes. Obesity synergizes with alcohol consumption in triggering the continuous progression of liver damage. Current consensus promotes a change in nomenclature from NAFLD to ‘metabolic associated fatty liver disease’ (MAFLD), to reflect also the associated metabolic abnormalities present in the disease (insulin resistance/type 2 diabetes and metabolic syndrome) [84]. NAFLD, affecting a quarter of the population, is the most common cause of liver diseases. Current studies suggest that hepatic cholesterol accumulation and changes in its regulation are important for the pathogenesis of NAFLD. Original data suggests that hepatic free cholesterol (FC) is an important lipotoxic molecule critical in the development of NASH, although the primary molecular mechanisms responsible for the buildup of fibrosis and inflammation that distinguish progressive NASH remain unclear [85]. Moreover, there is reliable evidence for a fundamental role of mitochondrial dysfunction in NASH pathophysiology, for review see [86]. Impaired mitochondrial function is involved at various levels in the pathogenesis of NASH since it increases oxidative stress and cytokine production, causing cell death, fibrosis, and inflammation. As a result, diminished ATP synthesis and increased ROS production have been described in the livers of NASH patients [87,88,89]. These biochemical changes are accompanied by ultrastructural abnormalities with the presence of a lesser number of mitochondria that appear bloated and round, with the presence of paracrystalline inclusions and loss of cristae [90,91]. In fact, and similar to what happens in ALD, increased mitochondrial FC reduces the fluidity of the mitochondrial membrane and compromises the function of the OGC carrier [41], thus depleting mGSH and favoring the generation of mitochondrial ROS [42]. TNF is found overexpressed in the liver and in the adipose tissue of NASH patients. This overexpression is more elevated in patients with more advanced NASH, corroborating that the TNF system is involved in the pathogenesis of NASH [92]. Thus, the functional consequences of mGSH depletion in NAFLD are sensitization of hepatocytes to TNF, permeabilization of mitochondrial membrane, cytochrome c release, hepatocyte necrosis, and apoptosis, which promote and perpetuate hepatic inflammation and cause NASH progression [42]. While the importance of mGSH has been clearly assessed in vitro and in experimental models, there are almost no studies evaluating this particular factor in patients. A pilot study recently examined the therapeutic effects of oral administration of GSH in patients with NAFLD. In this group of patients, following treatment with GSH for 4 months, ALT levels significantly decreased, and triglycerides, non-esterified fatty acids, and ferritin levels also decreased demonstrating the potential therapeutic effects of oral administration of GSH in practical dose for patients with NAFLD [93]. In parallel, an interesting and innovative study in patients with NAFLD aimed to elucidate the molecular mechanisms underlying the disease enlisted 86 subjects with variable grades of hepatic steatosis (HS) and acquired experimental data on lipoprotein fluxes. These data were used as personalized restrictions of a metabolic model genome-scale in hepatocytes to examine hepatic metabolic differences, with regards to its relations with other tissues. Their analysis predicted that subjects with elevated HS have an altered demand for GSH and NAD^+^. In addition, their metabolomics data exhibited that HS negatively correlated with plasma levels of glycine, serine, and associated metabolites, therefore suggesting that these precursors of GSH metabolism could be limiting [94]. In fact, an altered de novo GSH synthesis was proposed upon quantification of the hepatic expression levels of the associated enzymes. The addition of precursors for GSH and NAD^+^ biosynthesis to an experimental model in mice fed a Western diet prevented HS, thus confirming their findings. Additionally, in a proof-of-concept human study, they found enhanced liver function and reduced HS after supplementation with serine (a precursor of glycine) and therefore proposed a strategy for the treatment of NAFLD treatment [94]. These two studies highlight the relevance of maintaining proper GSH levels in NAFLD. However, large-scale clinical trials are needed to verify oral GSH efficacy, or of its precursors. It would be also interesting to see how this impacts mGSH levels and mitochondrial functionality.

### 4.3. Neurodegenerative Disorders

Mitochondrial dysfunction and oxidative damage are underlying many neurodegenerative disorders such as Alzheimer’s disease, Amyotrophic lateral sclerosis, Friedreich’s ataxia, Huntington’s disease, Multiple sclerosis, and Parkinson’s diseases, which point to mitochondrial oxidative stress as a causative factor of neurodegeneration [95]. 

In Alzheimer’s disease (AD), both amyloid-beta (Aβ) and hyperphosphorylated microtubule-associated protein tau (MAPT/TAU), the two main pathological hallmarks of AD, accumulate in mitochondria resulting in functional impairment and ROS generation [96,97]. Furthermore, studies using cell lines and mouse models harboring genetic mutations linked to familial AD, have demonstrated that a compromised mitochondrial antioxidant defense, unable to handle mitochondrial-derived ROS, promotes the amyloidogenic pathway and the activity of MAPT/TAU-related kinases, thus contributing to establish a vicious cycle of oxidative stress and damage [44,98,99]. In particular, cholesterol-mediated mGSH depletion associated with higher susceptibility to Aβ toxicity has been observed in isolated mitochondria from brains and cortical neurons of transgenic mice overexpressing active SREBP-2/sterol regulatory element binding transcription factor 2 (SREBF2) or Niemann-Pick type C1 (NPC1) knock-out mice, both animal models displaying enhanced intracellular FC levels [45,100]. Similarly, pharmacological reduction of the mitochondrial pool of GSH sensitized human neuronal and glial cell lines to Aβ-mediated cell death. Of therapeutic interest, neuroinflammation and neuronal damage were enhanced in transgenic SREBP-2 mice intracerebroventricular (icv) infused with Aβ and prevented upon mGSH recovery by GSH ethyl ester (GEE) coinfusion, which can diffuse through mitochondrial membranes, with a similar protection observed by intraperitoneal administration of GEE [45]. Accordingly, an AD mouse model that express mutant amyloid precursor protein (APP) and presenilin 1 (PS1) together with SREBP-2/SREBF2 displayed enhanced Aβ accumulation and tau pathology, correlating with early oxidative damage and neuroinflammation [44]. All these pathological alterations were prevented after in vivo GEE treatment [44]. More recently, enhanced Aβ-induced mitochondrial oxidative stress linked to cholesterol-mediated depletion of mGSH has also been shown to disrupt key mechanisms of cellular clearance resulting, contributing to Aβ deposition [101,102]. Thus, mitochondrial cholesterol accumulation emerges as a novel pathogenic factor in AD and the maintenance of mGSH levels as a potential target of therapeutic intervention.

Disruption of mitochondrial function is a key factor in Parkinson’s disease (PD) pathogenesis. The selective degeneration and loss of dopaminergic neurons in the substantia nigra of the ventral mid brain lead to dopamine depletion in the striatum causing oxidative stress and mitochondrial damage. These changes are restricted to the degenerating brain regions in PD and determine regional vulnerability [103]. Of note, mitochondrial impairment occurs early in PD pathogenesis, especially at the level of complex I, and animal models of PD are generated after administration of complex I inhibitors such as 1-methyl-4-phenylpyridinium (MPP+) [104]. Relevantly, familial PD is mainly characterized by mutations in genes involved in mitochondrial dysfunction, such as Parkin, α-synuclein, and leucine-rich repeat kinase 2 (LRRK2) [103,105,106,107]. On the other hand, exposure to pesticides that disrupt the mitochondrial function increases the likelihood of developing PD [108]. Loss of glutathione in the substantia nigra (30–50%), associated with a high proportion of oxidized glutathione, is a prominent hallmark of PD [109,110,111,112,113,114], and precedes the reduction of respiratory complex I activity and low dopamine levels [115]. Altogether, these findings suggest that therapeutic strategies directed to increase GSH levels may be of clinical significance. In this line, in vitro pretreatment with GEE has been shown to exert a protective effect in neurons directly exposed to H_2_O_2_ or incubated with respiratory complex I and II inhibitors MPP+ and malonate, respectively. In addition, in vivo studies in animal models of PD elevation of brain GSH by icv infusion of GEE have been reported to provide neuroprotection against oxidative stress caused by chronic mitochondrial impairment due to central delivery of MPP+ [116]. 

Amyotrophic lateral sclerosis (ALS) is characterized by a progressive degeneration of motor neurons in the brain and spinal cord. Approximately 10% of ALS cases are considered familial, while the other 90% are characterized as sporadic. A tight genetic linkage has been reported between familial ALS and the gene that encodes the Cu/Zn-binding superoxide dismutase (SOD1), a metalloenzyme that catalyzes the dismutation of the superoxide anion (O_2_^●−^) to O_2_ and H_2_O_2_. Nearly 150 mutant forms of SOD1 have been identified in ALS patients, which are responsible for approximately 20% of all the inherited cases [117]. In the rest, although etiology remains still unknown, oxidative damage associated with mitochondrial dysfunction has been shown to play a contributive role [118,119]. Depletion of GSH underlies progression of ALS. Thus, strategies aimed at elevating GSH may yield new therapeutics for ALS. In this regard, a recent study evaluated the use of a nutritional cystine-rich GSH precursor, whey supplement (Immunocal (^®^)), in the mutant hSOD1 (G93A) mouse model of ALD [120]. The administration of the GSH precursor significantly delayed the disease onset in the transgenic hSOD1(G93A) mice, but without extending life span, most likely due to the inability to recover the mitochondrial GSH pool in the spinal cord [120]. 

Of relevance for neurodegenerative disorders, a recent study has uncovered that the GSH redox pathway regulates mitochondria dynamics in axons [121]. Specifically, the study in *Drosophila* identifies a novel glutathione S-transferase (GST), Gfzf, homologous to GSTT1 in humans, that inhibits mitochondrial hyperfusion under normal physiological conditions. The authors show that changes in the redox balance due to GST loss have a direct impact on mitochondrial trafficking and neuronal response. Remarkably, genome-wide association studies (GWAS) have linked polymorphisms in GST genes [122] to increased risk in developing AD and PD later in life. Future studies will be needed to analyze whether changes in the GSH:GSSG ratio associated to GST activity can alter mitochondrial dynamics described in neurodegenerative disorders, which will provide new mechanistic insights into how these alterations result in an axonal loss.

### 4.4. Diabetic Nephropathy

Mitochondrial ROS generation is exacerbated during diabetes either by alterations in oxidative phosphorylation, by antioxidant depletion, or both [123,124,125,126]. In turn, antioxidant depletion, particularly of GSH, may favor peroxidative damage in lipids from mitochondrial membranes [127,128]. These oxidative injuries have a direct effect in the mitochondrial electron transfer, resulting in an enhanced electron leak and ROS generation that lead to a vicious circle of mitochondrial dysfunction and oxidative stress. Diabetic nephropathy (DN), referring to the deterioration of kidney function associated to both Type 1 and Type 2 diabetes, can progress to chronic kidney disease and, in fact, is a strong predictor of mortality in diabetic patients. Emerging evidence points to the oxidative and nitrosative stress as the underlying mechanism by which chronic hyperglycemia causes renal cellular damage [129,130,131,132]. Low levels of renal GSH have been described associated with DN [131,133,134]. In turn, dietary GSH supplementation has been shown to partially protect against many of the pathological changes due to DN [135]. Thus, given these findings, it would be logical to suggest that the mGSH pool may be a good choice as a therapeutic target. Of interest, a very recent study using the delivery of GSH to the kidney with liposomal technology (GSH-LIP) revealed that the complex with liposomes improves the bioavailability and antioxidant capacity of GSH to scavenge redundant ROS induced by oxidative stress. Furthermore, in vivo imaging showed that GSH-LIP is directed to the kidney and significantly recovers renal function. Thus, these studies provide the foundation for study the use of antioxidant-related drugs in DN [136].

### 4.5. Aging and Age-Related Diseases

Aging is a time-sequential degradation of cellular functions caused by accumulated damage that leads to organ failure, and finally death. A wide number of aging theories have been proposed over the years, but the mitochondrial free radical theory of aging is still the best theoretical framework to explain aging and longevity in mammals [137]. According to this theory, aging is characterized by the loss of redox homeostasis associated with a reduction of the detoxification capacity of cells, which correlates with an increased risk for age-related diseases [138,139]. Mitochondrial dysfunction and decay have been widely related to aging and age-related diseases, such as neurodegenerative disorders [140,141]. Increased oxidant leakage, mitochondrial DNA damage, and susceptibility to apoptotic pore formation are all features displayed by mitochondria from aged tissues [142]. Particularly important for mitochondrial fitness is the role of mGSH in the regulation of ATP production. Critical protein sulfhydryl redox states depend on mGSH levels, which in turn influence both NADH and FADH2 generation and the electron flow through the ETC [24,143,144]. mGSH decreases up to 50% with age [145,146], being more marked in male than female mice in many tissues. This age-related depletion of mGSH content may be attributable to different factors such as an enhanced use due to an increasingly oxidant-rich environment or a defective mitochondrial transport resulting from a progressive loss of the mitochondrial membrane fluidity, and it may also reflect a lower rate of synthesis [147]. In addition, both plasma glutathione and cysteine, a key precursor amino acid for GSH synthesis become oxidized with aging [139]. It has also been suggested that the mortality and frailty risk in the elderly associated to low dietary protein intake is mainly due to low cysteine availability [148], and that dietary supplementation with cysteine and glycine by promoting GSH synthesis could be notably protective against oxidative stress associated to aging [149]. A proof of concept for the causal role of GSH in the aging process is the fact that over-expression of the enzyme GCS has been shown to prolong the life span of Drosophila by up to 50% [150]. In addition, it is known that the number of mitochondria decreases with age in liver cells of mice [151], rats [152,153], and humans [154,155], concurrent with a decrease in mitochondrial DNA copy number and mitochondrial protein levels [156].

As we have seen, the importance of mGSH has been illustrated by the emergent number of pathologies in which its decrease below a threshold produces cell damage, even leading to cell death. This is why modulation of mGSH levels can influence disease progression, and therapies aimed at recovering mGSH levels may be of medical importance in numerous human diseases.

### 4.6. Others

The amount of human pathologies or clinical settings in which mGSH may be playing a critical role is still growing. In fact, any situation where a mitochondrial source of ROS is detected, either directly such as drugs interacting with subunits of the respiratory chain, or indirectly as in defective mGSH carriers due to lipid changes in the mitochondrial membrane, mGSH could be responsible for cell demise. Recent data point to different topics for future analysis of mGSH involvement, such as:

▪ Lung diseases—glutathione precursors, particularly N-acetylcysteine, have been prescribed for years to prevent acute pulmonary episodes, bronchitis, or emphysema as an effective method of reducing oxidative stress in clinical settings associated with low GSH levels, such as chronic lung diseases [157]. More recently, glutathione levels have been associated with COVID-19 disease. Since mitochondrial ROS act as signal-transducing molecules that upregulate inflammatory cytokines [158] in conditions with excessive inflammatory response, as happens in severe COVID-19 symptoms, it is expected that mitochondrial antioxidants such as mGSH would play a role during the Severe acute respiratory syndrome coronavirus 2 (SARS-CoV-2) viral infection.
▪ Chemotherapy—standard chemotherapeutic agents such as doxorubicin and cisplatin are well-known inducers of mitochondrial ROS during their anti-tumoral action [159,160]. However, the mitochondrial effects of other more recently approved anti-cancer drugs, such as sorafenib or regorafenib, are being just revealed [161,162]. More importantly, mitochondrial antioxidants may reduce the effectivity of these drugs, while glutathione depressors potentiate their effect in hepatocellular carcinoma cells (Cucarull et al., unpublished results). Since chemotherapy-induced side effects are frequently also caused by mitochondrial ROS, such as cardiotoxicity or kidney injury after doxorubicin and cisplatin treatments, respectively, these potential redox therapies should be carefully directed to the target cells. Therefore, intake of antioxidants or ROS modulators should be well controlled in order to avoid undesired effects during cancer treatment.
▪ Gender perspective—GSH levels are different in males and females as a consequence of hormonal regulation and aging [163,164]. Although higher mGSH levels in females are expected, this topic has been poorly pursued, with very few studies in animal models and common pathologies. Novel results highlighting the antioxidant differences observed between sexes, frequently reflecting sexual dimorphism in disease incidence will increase the interest in specific mGSH levels and maybe suggest gender-specific biomedical strategies.

## 5. Conclusions

mGSH plays a center role in the cellular defense from death by being a key regulator of mitochondrial oxidative stress. However, up to now, although our knowledge of mitochondrial redox control systems has increased notably, we still lack the full understanding of how mGSH transport works in the different cells/organs. Although several carriers have been identified, they most probably do not account for the totality of mGSH transport. In addition, there are conflicting data regarding the acknowledged role of the known mGSH carriers (DIC and OGC) in the transport of GSH, which need to be addressed. Thus, more effort is needed in the discovery and characterization of these mGSH carriers. Numerous pathologies course with mGSH depletion, being in most cases a causative factor for disease progression. Accordingly, novel strategies aimed either at preventing mGSH depletion, such as mitochondrial cholesterol lowering agents for liver pathologies, or drugs capable of increasing mGSH levels need to be pursued from a therapeutic perspective. 

## Abbreviations

2-oxoglutarate carrier (OGC; SLC25A11); adenine nucleotide translocator (ANT); alcoholic liver disease (ALD); alcoholic steatohepatitis (ASH); Alzheimer’s disease (AD); amyloid beta peptides (Aβ); amyloid precursor protein/presenilin 1 (APP/PS1); amyotrophic lateral sclerosis (ALS); Cu/Zn-superoxide dismutase (SOD1); diabetic nephropathy (DN); electron transport chain (ETC); endoplasmic reticulum (ER); free cholesterol (FC); glyoxalase II (GLO2); Glutathione (GSH); glutathione disulfide (GSSG); glutathione reductase (GR); glutathione S-transferase (GST); GSH ethyl ester (GEE); GSH peroxidases (GPx); hepatic steatosis (HS); metabolic associated fatty liver disease (MAFLD); mitochondrial dicarboxylate carrier (DIC; SLC25A10); mitochondrial glutathione (mGSH); mitochondrial NADP+-dependent isocitrate dehydrogenase (IDPm); mitochondrial permeability transition (MPT); non-alcoholic fatty liver disease (NAFLD); non-alcoholic steatohepatitis (NASH); Parkinson’s disease (PD); peroxiredoxins (Prx); protein disulfide isomerase (PDI); reactive oxygen species (ROS); S-adenosyl-l-methionine (SAM); S-d-lactoylglutathione (SLG); sterol regulatory element binding protein 2 (SREBP-2); thioredoxin 2/thioredoxin reductase 2 (Trx2/TrxR2); tumor necrosis factor (TNF).
